# Novel O-palmitolylated beta-E1 subunit of pyruvate dehydrogenase is phosphorylated during ischemia/reperfusion injury

**DOI:** 10.1186/1477-5956-8-38

**Published:** 2010-07-09

**Authors:** Clifford DL Folmes, Grzegorz Sawicki, Virgilio JJ Cadete, Grant Masson, Amy J Barr, Gary D Lopaschuk

**Affiliations:** 1 Cardiovascular Research Group and the Departments of Pharmacology and Pediatrics, The University of Alberta, Edmonton, Alberta, Canada; 2 Department of Pharmacology, University of Saskatchewan, Saskatoon, Saskatchewan, Canada; 3 Department of Clinical Chemistry, Medical University of Wroclaw, Wroclaw, Poland

## Abstract

**Background:**

During and following myocardial ischemia, glucose oxidation rates are low and fatty acids dominate as a source of oxidative metabolism. This metabolic phenotype is associated with contractile dysfunction during reperfusion. To determine the mechanism of this reliance on fatty acid oxidation as a source of ATP generation, a functional proteomics approach was utilized.

**Results:**

2-D gel electrophoresis of mitochondria from working rat hearts subjected to 25 minutes of global no flow ischemia followed by 40 minutes of aerobic reperfusion identified 32 changes in protein abundance compared to aerobic controls. Of the five proteins with the greatest change in abundance, two were increased (long chain acyl-coenzyme A dehydrogenase (48 ± 1 versus 39 ± 3 arbitrary units, n = 3, P < 0.05) and α subunit of ATP synthase (189 ± 15 versus 113 ± 23 arbitrary units, n = 3, P < 0.05)), while two were decreased (24 kDa subunit of NADH-ubiquinone oxidoreductase (94 ± 7 versus 127 ± 9 arbitrary units, n = 3, P < 0.05) and D subunit of ATP synthase (230 ± 11 versus 368 ± 47 arbitrary units, n = 3, P < 05)). Two forms of pyruvate dehydrogenase βE1 subunit, the rate-limiting enzyme for glucose oxidation, were also identified. The protein level of the more acidic form of pyruvate dehydrogenase was reduced during reperfusion (37 ± 4 versus 56 ± 7 arbitrary units, n = 3, P < 05), while the more basic form remained unchanged. The more acidic isoform was found to be O-palmitoylated, while both isoforms exhibited ischemia/reperfusion-induced phosphorylation. *In silico *analysis identified the putative kinases as the insulin receptor kinase for the more basic form and protein kinase Cζ or protein kinase A for the more acidic form. These modifications of pyruvate dehydrogenase are associated with a 35% decrease in glucose oxidation during reperfusion.

**Conclusions:**

Cardiac ischemia/reperfusion induces significant changes to a number of metabolic proteins of the mitochondrial proteome. In particular, ischemia/reperfusion induced the post-translational modification of pyruvate dehydrogenase, the rate-limiting step of glucose oxidation, which is associated with a 35% decrease in glucose oxidation during reperfusion. Therefore these post-translational modifications may have important implications in the regulation of myocardial energy metabolism.

## Background

Myocardial ischemia results from the transient blockage of the systemic circulation due to a number of mechanisms including atherosclerosis, embolism and surgical procedures resulting in a mismatch between the oxygen requirement of the heart and the oxygen supplied via the coronary circulation. Although many studies have examined ischemia/reperfusion (I/R) injury and possible therapeutic strategies, the exact molecular and cellular mechanisms continue to be elusive and it remains devastating cause of morbidity and mortality worldwide. Proteomics provides a powerful experimental approach for the observing the global changes in protein abundance and has recently been used to identify novel post-translational modifications (PTMs) of these proteins [1,2]. Utilizing this approach, several studies have examined the I/R and preconditioning induced changes of the heart proteome [3-8]. Interestingly, many of the identified proteins have important functions in cardiac energy metabolism, which suggests an important role for alterations in metabolism as a contributor to the pathophysiology of I/R injury.

Under normal aerobic conditions, the heart preferentially metabolizes fatty acids, which contribute between 60% and 80% of the required ATP [9]. However, during reperfusion fatty acid oxidation quickly recovers, at the expense of glucose oxidation, and predominates as the main source of mitochondrial oxidative metabolism [10-12]. This is believed to be due to the exposure of the heart to high levels of circulating fatty acids, as well as to subcellular changes in the control of myocardial fatty acid oxidation [13,14]. In addition, anaerobic glycolysis increases and becomes an important source of ATP for the maintenance of ion gradients in the cardiomyocyte during ischemia [15]. However, if the pyruvate from glycolysis is not subsequently oxidized, and glycolytically derived ATP is hydrolyzed, there is a net production of both lactate and protons due to the uncoupling of glycolysis from glucose oxidation [16,17]. The protons produced are a major contributor to the intracellular acidosis that is associated with ischemia. This acidosis can lead to adverse events, including accelerated sarcolemmal Na^+^/H^+ ^exchange resulting in intracellular Na^+ ^and Ca^2+ ^overload [18], the initiation of cardiac arrhythmias [19,20] and a decreased response of contractile proteins to Ca^2+ ^[19]. If the pyruvate from glycolysis is aerobically metabolized (i.e. glucose oxidation), then lactate and protons are not produced, leading to improved functional recovery during reperfusion [21].

The majority of the heart's essential catabolic machinery resides in the mitochondria. Despite the fact that the human heart mitochondrial proteome has been resolved and approximately 50% of the identified proteins are involved in metabolism [22], only a few studies has specifically examined the I/R induced global changes in the mitochondrial proteome [7,8]. In this study we used a proteomics approach to observe the I/R induced changes in abundance of mitochondrial proteins, one of which was identified as the βE1 subunit of pyruvate dehydrogenase (PDH_βE1_), a key regulatory enzyme that directly modulates the rates of glucose oxidation. We subsequently demonstrated that this subunit contains novel post-translational modification (PTMs). These PTMs may lead to the changes in enzyme activity, and are therefore potentially sites regulating myocardial energy metabolism during ischemia and reperfusion.

## Methods

### Animals

The University of Alberta adheres to the principles for biomedical research involving animals developed by the Council for International Organizations of Medical Sciences and complies with Canadian Council of Animal Care guidelines.

### Isolated working rat hearts

Rat hearts were cannulated for isolated working heart perfusions as described previously [23]. 9 biological replicates per group were utilized for cardiac function with 6 of these biological replicates utilized for measurement of myocardial metabolism and 3 biological replicates utilized for subsequent 2-D PAGE analysis. In brief, male Sprague-Dawley rats (0.25-0.3 Kg) were anesthetized with sodium pentobarbital (60 mg/Kg i.p.), and the hearts were quickly excised, and the aorta and left atria cannulated. Oxygenated Krebs-Henseleit solution containing 1.2 mM palmitate bound to 3% BSA, 5 mM glucose, and 100 μU/mL insulin was delivered to the left atrium at a preload pressure of 11.5 mmHg. One series of hearts was perfused for 35 min of aerobic perfusion, 25 min of global no-flow ischemia, followed by 40 min of aerobic reperfusion. This time of ischemia results in myocardial stunning without significant myocardial necrosis and was chosen to result in approximately 30% recovery of mechanical function during reperfusion. Another series of hearts underwent 100 min of aerobic perfusion to serve as a time-matched aerobic control group. These hearts were immediately removed after the perfusion protocol and mitochondria were isolated using a differential centrifugation protocol previously described [24]. Cardiac work was calculated as the product of aortic systolic pressure, measured by a Gould P21 pressure transducer (Harvard Apparatus) connected to the aortic outflow line, and cardiac output, measured with a T206 ultrasonic flow probe (Transonic Systems Inc.) in the preload line.

### Measurement of glucose and palmitate oxidation

An additional series of hearts were perfused using the I/R protocol to measure rates of myocardial energy metabolism. Glucose oxidation and palmitate oxidation were measured by perfusing hearts with [U-^14^C]glucose or [9,10-^3^H]palmitate, respectively, with quantitative measurement of ^14^CO_2 _or ^3^H_2_O production, as described previously [23].

### Isolation of Mitochondria

After the perfusions, the hearts were quickly rinsed in ice-cold 225 mM mannitol, 75 mM sucrose, 1 mM EGTA, 10 mM Tris, pH 7.5 (MSE buffer). The atria were removed and the ventricles minced. A 20% (w/v) tissue suspension in MSE buffer was adjusted between pH 7.4 to 7.5. The suspension was homogenized with a Polytron homogenizer for two 10-second periods. The homogenate was centrifuged at 480 × g for 5 min, and the supernatant (S1) was filtered through cheese cloth and centrifuged at 10,000 × g for 30 min. The pellet (Pi), from the 480 × g spin, was suspended in MSE buffer and homogenized again under the same settings and centrifuged at 480 × g for 5 min. The supernatant (S2) was filtered through cheesecloth and was centrifuged at 10,000 × g for 30 min. The S2 pellet was combined with the S1 pellet suspended using MSE buffer and centrifuged at 10,000 × g for 30 min. The resulting pelleted mitochondria (S3) was suspended in a urea solution in a 1:2 (w/v) ratio and used for 2D electrophoresis [24]. Mitochondrial purity was assessed by Western blotting for the cytosolic marker, GAPDH (Abcam) and mitochondrial marker, mitochondrial creatine kinase (mtCK, Abcam).

### Preparation of mitochondria extracts

Protein samples for 2-D electrophoresis were prepared at room temperature by mixing one part of the mitochondria pellet with two parts (w:v) of rehydration buffer (8 mol/L urea, 4% CHAPS, 10 mmol/L DTT, 0.2% Bio-Lytes 3/10 [BioRad]). Samples were sonicated twice for 5 s and centrifuged for 10 min at 10,000 g at room temperature to remove any insoluble particles. Protein content of the mitochondrial extract in rehydration buffer was measured using the BioRad protein assay.

### Two-dimensional polyacrylamide gel electrophoresis

400 μg of mitochondria extract protein was originally applied to 11 cm immobilized pH gradient strip with linear pH gradient from 3 to 10 (IPG, Biorad) and equilibrated for 16-18 hr at 20°C in rehydration buffer, however we subsequent utilized a pH gradient of 5 to 8 to increase the resolution and separation of protein spots. These conditions have been previously validated to result in even protein loading [25]. For isoelectrofocusing, the BioRad Protean isoelectrofocusing cell was used with the conditions described previously [26]. The second dimension of electrophoresis was carried out using 8-16% acrylamide Criterion precast gradient gels (BioRad). To minimize variations in resolving proteins during the second dimension run, all gels were run simultaneously using a Criterion Dodeca Cell (BioRad). After separation, proteins were detected using Coomassie Brilliant Blue R-250 (BioRad). Developed gels were scanned using a calibrated GS-800 densitometer (BioRad). Quantitative analysis of spot intensity from 2-D electrophoresis was measured using PDQuest 7.1 software (BioRad). Only protein spots with a relative intensity between 10-100 arbitrary units were considered for analysis. Using these criteria for protein resolution and staining, we were able to obtain high reproducibility to analyze both a single protein from the same sample run in different gels and for a specific protein spot from different heart samples. 3 analytical replicates were utilized for 2-D PAGE and subsequent mass spectrometry identification.

### Mass spectrometry

Protein spots were manually excised from the 2-D gel. Subsequently, in-gel trypic digests were analyzed using a Bruker Ultraflex TOF/TOF mass spectrometer. Digests were applied to an AnchorChip plate (Bruker) as previously described [27]. Mass spectra and tandem mass spectra were then obtained in an automated fashion using the AutoXecute function. Typically 100 shots were accumulated to generate a peptide mass fingerprint. The 5 most intense peaks were then selected to have MS/MS performed where 400 shots on average were used to generate a MS/MS profile (3 analytical replicates). Peaklist generation and peak picking was performed using Flexanalysis 2.2 SNAP (sophisticated numerical annotation procedure), with default setting for peaklist generation. For PMF data the S/N threshold = 6, resolution >6000, they were externally calibrated using 8 peptides with m/z ranging from 757.39 to 3147.47 and tryptic autolytic peptides are excluded. Acceptance criteria for MS/MS was at least 2 matching peptides with a score P < 05. The resultant MS or MS/MS data was searched using the MASCOT version 2.2 http://www.matrixscience.com search engine against NCBInr (2007) and Swiss-Prot (2007) databases. Specified 68,229 protein sequences from NCBInr for Rattus Norvegicus was used for identification of the proteins. The Mowse scoring algorithm was used for justification of protein identification [28].

### Calculation of the theoretical masses of PDHβ peptides generated by the enzymatic cleavage

PeptideMass http://au.expasy.org/tools/peptide-mass.html was used to predict the mass of trypsin-digested peptides from the sequence of PDHβ. For calculation of theoretical peptide masses we assumed: 1) incomplete protein digestion (1 missed cleavage level) and ± 0.2 Da error windows on experimental peptide mass values and for MS/MS fragment ion mass values, 2) mandatory alkylation and reduction of cysteines with iodoacteamide, and 3) variable oxidation of methionine. Only peptides bigger than 500 Da were considered.

### Examination of experimental peptide mass fingerprinting for post-translational modifications (PTMs)

FindMod tool (2007) was used to find potential PTMs http://au.expasy.org/tools/findmod/ in the experimental peptides of PDHβ. 22 types of PTMs were considered with the assumption that up to 3 PTM in one peptide can exists.

### Chemical verification of O-palmitolyation

Experimental peptides with identified (*in silico*) palmitoylation were incubated with 0.1 M KOH for 4 hr at room temperature [29] and analysed by MALDI TOF-TOF.

### Verification of phosphorylation by dephosphorylation of peptide with alkaline phosphatase

Experimental peptides with identified (*In silico*) phosphorylation were incubated with 0.5 unit of alkaline phospatase in 50 mM bicarbonate solution for 120 min at 30°C and analyzed by MALDI TOF-TOF [30].

### Prediction of kinases that phosphorylate the βE1 PDH

PDH phosphorylating kinases were predicted using phosphorylation consensus sequences identified with Scansite http://scansite.mit.edu/ and NetPhosK 1.0 Sever http://www.cbs.dtu.dk/services/NetPhosK/.

### Western blotting

Protein (30 μg) from heart homogenate or from mitochondria preparation was separated using 12% SDS-PAGE and transferred to a PVDF membrane (Bio-Rad). Glyceraldehydes 3-phosphate dehydrogenase (GAPDH) was identified using mouse monoclonal anti-GAPDH antibody (Abcam). Mitochondrial creatine kinase (mtCK) was identified with rabbit polyclonal anti-mtCK antibody (Abcam). Band densities were measured using Versa Doc 5000 and Quantity One 4.6 software (Bio-Rad).

### Statistical analysis

Data are shown as mean ± SEM. Analysis of functional data, the protein spots in the 2-D electrophoresis experiments and metabolic data were performed using unpaired t-tests. A value of p < 0.05 was considered statistically significant.

## Results

### Cardiac function in hearts subjected to aerobic perfusion and I/R

**Figure 1:**
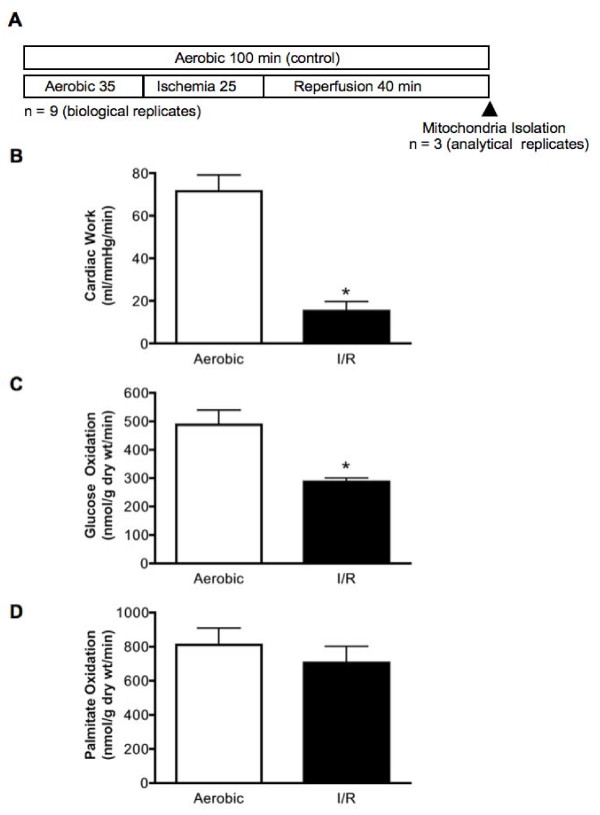
**Ischemia and reperfusion reduced cardiac work compared to aerobic control isolated working hearts**. (A) Experimental protocol of hearts subjected to I/R. Arrow indicates when the mitochondrial isolation began. (B) Mechanical function of the heart after I/R injury. (C) Rates of glucose oxidation in isolated working rat hearts subjected to aerobic perfusion or I/R. (D) Rates of palmitate oxidation in isolated working rat hearts subjected to aerobic perfusion or I/R. Values represent the mean ± SEM of 9 hearts per group for mechanical function and 6 hearts per group for metabolic rates.* represents P < 0.05, significantly different from aerobic value.

Hearts were perfused either for 100 min aerobically, or for 35 min aerobically followed by 25 min of global ischemia and 40 min of reperfusion (Figure 1A). The functional recovery of hearts subjected to I/R was significantly reduced compared to hearts aerobically perfused for 100 min (Figure 1B). Glucose oxidation rates were decreased by 35% in reperfused hearts compared to the average glucose oxidation rates in time-matched aerobic control hearts (Figure 1C). Despite this reduction in glucose oxidation rates, palmitate oxidation rates did not differ between the I/R group and the aerobic controls (Figure 1D).

### 2-D Electrophoresis and identification of proteins

**Figure 2:**
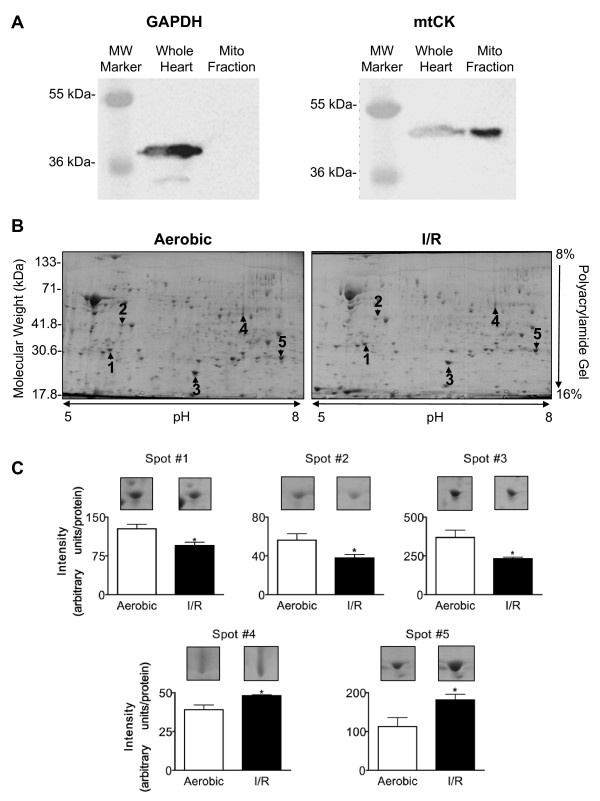
**2-D electrophoresis identified five cardiac mitochondrial proteins that are modified following ischemia and reperfusion**. (A) Western blots for GAPDH and mtCK to assess the purity of the mitochondrial preparation from aerobic hearts. (B) Representative 2-D electrophoresis of heart mitochondria indicating spots picked for identification. (C) Quantification of spots and representative gels. Values represent the mean ± SEM of 3 hearts per group. * represents P < 0.05, significantly different from aerobic value.

**Figure 3:**
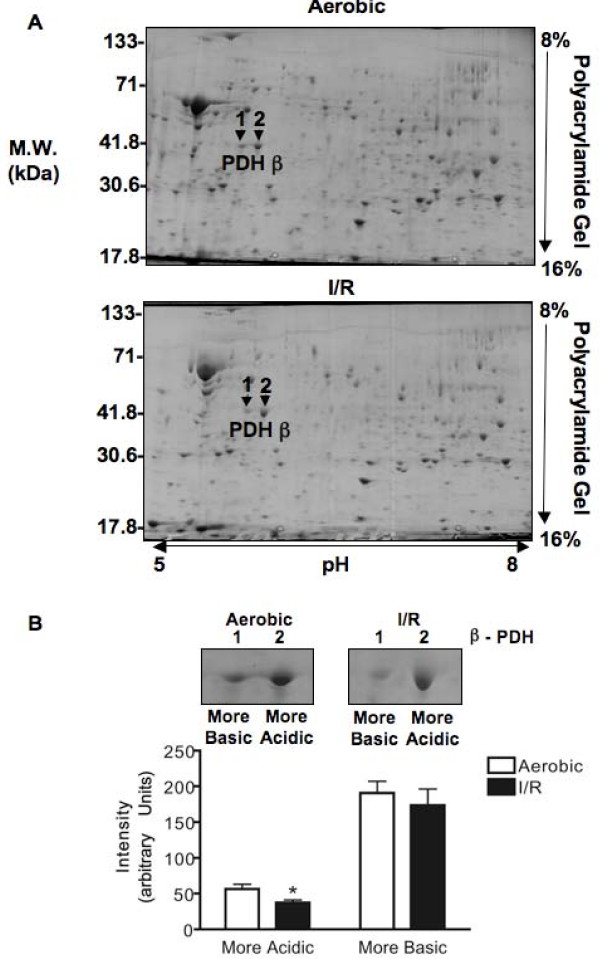
**2-D electrophoresis identified two forms of the βE1 of pyruvate dehydrogenase (PDH)**. (A) Representative 2-D electrophoresis of heart mitochondria indicating spots identified as βE1 subunit of PDH. (B) Quantification of spots identified as the βE1 of PDH peptides and representative gels. Values represent the mean ± SEM of 3 hearts per group. * represents P < 0.05, significantly different from aerobic value.

The mitochondrial isolation resulted in a similar yield of mitochondria (97 ± 8 and 102 ± 8 mg mitochondria/g wet heart weight) with enriched expression of mtCK and free of contamination by cytosolic GAPDH (Figure 2A). 2-D PAGE identified 32 I/R-induced changes in protein levels out of approximately 260 protein spots observed in all of the gels and we chose to identify the five spots with the greatest change in abundance (Figure 2B, C and Table 1). Mass spectrometry analysis of all five protein spots gave only one protein hit above the threshold for each search. The high Moswe scores show high level of accuracy for each identification (Table 1). Two of these protein abundances were increased with I/R and were identified as long chain acyl-coenzyme A dehydrogenase and ATP synthase (α subunit). Three of these protein abundances were decreased with I/R and were identified as NADH-ubiquinone oxidoreductase (24 kDa subunit), ATP synthase (D subunit) and PDH (βE1 subunit). In addition, the protein spot adjacent to spot 2 was also identified as PDH_βE1 _(Table 2). For the remainder of this study we concentrated on post-translational changes induced in PDH due to the importance of this enzyme in the regulation of myocardial glucose oxidation. Both aerobic and I/R groups expressed two molecular forms of PDH_βE1 _(which we identified as a more acidic form and more basic form based on their isoelectric point) but only the protein level of the more acidic form was decreased in I/R (Figure 3B).

**Table 1:** Mass Spectrometry identification of protein spots using the Mascot Search Engine.

Protein	Probability Based Mowse Score*		Peptides		Sequence	Identification and AC** (#)
Spot (#)	Threshold	Observed	Matched	Not matched	Coverage	
	(P < 0.05)	Score	(n)	(n)	(%)	
						NADH-ubiquinone
1	58	141	17	41	44	oxidoreductase (24 kDa subunit),
						P19234
						Pyruvate dehydrogenase
2	32	108	3	31	10	(β E1 subunit)
						P49432
						ATP synthase
3	58	127	13	40	34	(D subunit)
						P31399
4	58	127	13	18	34	Long-chain acyl-CoA dehydrogenase
						P15650
						ATP synthase
5	58	87	12	28	41	(α subunit)
						P15999

* -10 log(P) where P is the probability that the observed match is a random event.

**UniProtKB/Swiss-Prot primary accession number

**Table 2:** Mass Spectrometry identification of protein spots corresponding to the E1 subunit of PDH.

Protein	Probability Based Mowse Score*		Peptides		Sequence	Identification
Spot (#)	Threshold	Observed	Matched	Not matched	Coverage	
	(P < 0.05)	Score	(n)		(%)	
1 Aerobic	32	108	3	31	10	Pyruvate dehydrogenase (βE1 subunit)
2 Aerobic	69	167	21	5	59	Pyruvate dehydrogenase (βE1 subunit)
1 I/R	31	85	2	40	13	Pyruvate dehydrogenase (βE1 subunit)
2 I/R	69	151	21	13	59	Pyruvate dehydrogenase (βE1 subunit)

* -10 log(P) where P is the probability that the observed match is a random event.

### Protein mass fingerprints (PMFs) and modifications of PDH

**Figure 4:**
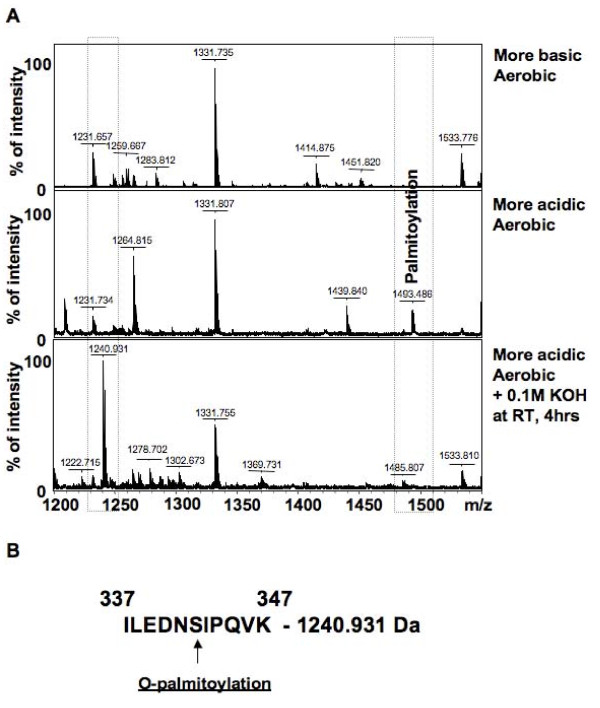
**Protein mass fingerprints from aerobic heart mitochondria showing the novel O-palmitoylation of the more acidic form of PDH**. (A) PMFs of the more basic and more acidic forms of the βE1 of PDH, and the PMF of the more acidic form of the βE1 after potassium hydroxide treatment (0.1M KOH room temperature for 4 hours). (B) Potential site of O-palmitoylation of the more acidic form of βE1.

Proteins were excised from the gels and subjected to trypsin digestion followed by mass spectrometry analysis to produce PMFs of both the more acidic and more basic form of the βE1 subunit of PDH (Figure 4A). Comparison of trypsin digested peptides of PDH_βE1 _from control hearts using the FindMod tool showed that the more acidic form is O-palmitoylated (peptide 1493.486 Da, Figure 4A and 4B). This unique PTM was confirmed by KOH treatment of the peptides (Figure 4A), which led to the disappearance of the O-palmitoylated peptide and the appearance of a new peptide (1240.931 Da).

**Figure 5:**
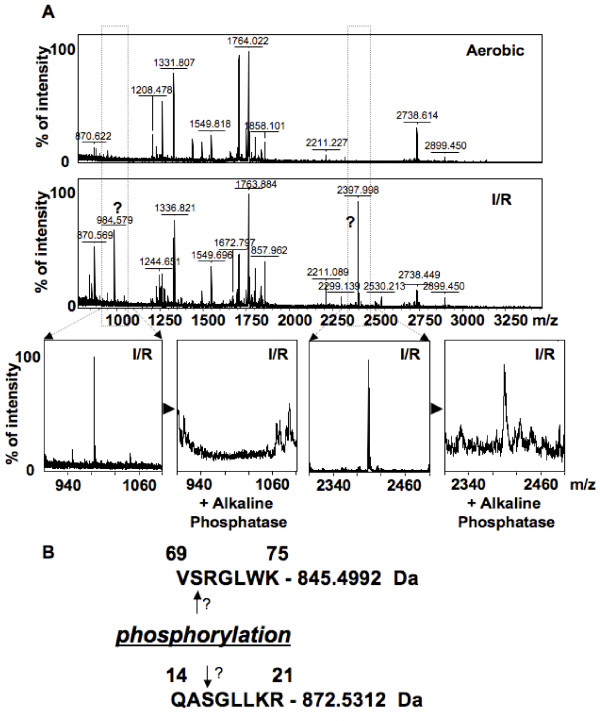
**Protein mass fingerprints of the more acidic form of PDH showing possible phosphorylation**. (A) PMFs of the more acidic from of the βE1 of PDH from aerobic and I/R heart mitochondria and the PMF after alkaline phosphatase treatment (2 hrs at 30°C in 50 mM NH_4_HCO_3_). (B) Potential site of phosphorylation of the more acidic form of the βE1 of PDH.

**Figure 6:**
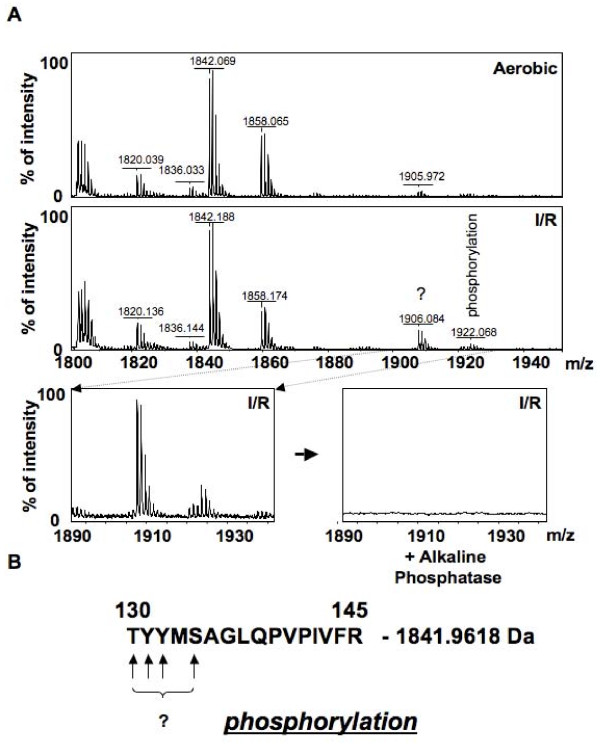
**Protein mass fingerprints of the more basic form of PDH showing possible phosphorylation**. (A) PMFs of the more basic from of the βE1 of PDH from aerobic and I/R heart mitochondria and the PMF after alkaline phosphatase treatment (2 hr at 30°C in 50 mM NH_4_HCO_3_). (B) Potential site of phosphorylation of the more basic form of the βE1 of PDH.

**Figure 7:**
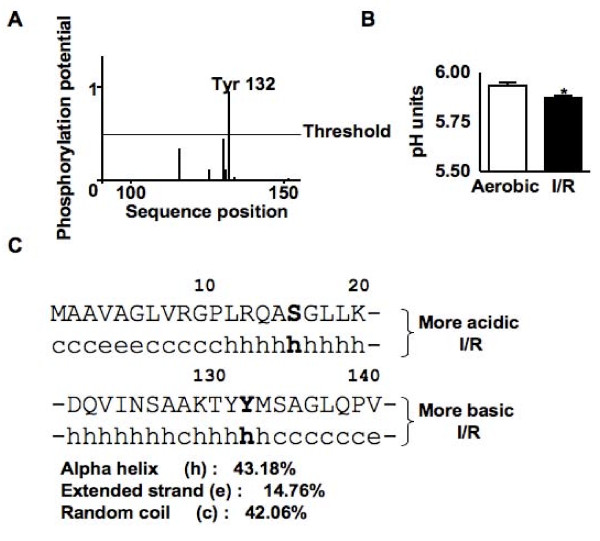
**Identification of putative kinases for the phosphorylation of the βE1 of PDH**. (A) Graph showing the phosphorylation potential of amino acids in the more basic form of the βE1 of PDH. (B) Isoelectric point of the more basic form of the βE1 of PDH. (C) Predicted secondary structure surrounding the putative phosphorylation sites.

Upon comparison of the PMFs from the more acidic form of PDH_βE1 _from the aerobic and I/R hearts, two new peptides with masses 984.579 Da and 2397.998 Da were detected as being putative phosphorylated peptides (Figure 5). However upon treatment with alkaline phosphatase, only the smaller peptide was removed (Figure 5). Comparison of the PMFs of the more basic form of PDH_βE1 _from the aerobic and I/R hearts with the Find Mod tool, detected that I/R triggers phosphorylation of the more basic form (peptide 1922.068, Figure 6A and 6B). This peptide peak disappeared after treatment with alkaline phosphatase (Figure 6A). In addition, the isoelectric point of the more basic form of PDH_βE1 _was decreased in I/R hearts (Figure 7B), further supporting the theory that this peptide was phosphorylated.

### Identification of putative phosphorylation sites and kinases

Phosphorylation sites were determined by examining the sizes of trypsin digested protein fragments with the FindMod tool, the kinases were determined using Scansite and the NetPhosK 1.0 Sever. Using this analysis we identified Ser-16 of the more acidic PDH_βE1 _to be a possible site of phosphorylation with the putative kinase being protein kinase Cζ (PKCζ) or protein kinase A (PKA) (Table 3). For the more basic PDH_βE1 _we identified Tyr-132 as a possible site of phosphorylation with the putative kinase being the insulin receptor kinase (Table 3).

**Table 3:** Identification of phosphorylation sites and putative kinases for the E1 subunit of PDH.

Isoform Of PDH	Amino Acid	Scansite		NetPhosK 1.0 Server	
		Kinase	Score*	Kinase	Score*
More Acidic	S16	PKCζ	0.61	PKA	0.57
More Acidic	S70			PKA	0.53
More Basic	T130	PKC			
More Basic	Y131				
More Basic	Y132	INSR	0.72	INSR	0.55
More Basic	S134				

* Threshold 0.5 (range 0-1)

## Discussion

Although there are several reports describing the mitochondrial proteome, only two previous studies have attempted to provide a picture of more global changes in protein abundance induced by I/R [7,8]. Using a functional proteomics approach we identified 32 protein spots, which are either up or down regulated by I/R. We focused on the proteins with the largest changes in abundance, which all corresponded to proteins with metabolic functions. The proteins with an increase in abundance included long chain acyl-coenzyme A dehydrogenase and ATP synthase (α subunit), while proteins with a decrease in abundance included NADH-ubiquinone oxidoreductase (24 kDa subunit), ATP synthase (D subunit) and PDH_βE1_. Due to the identification of two protein spots corresponding to PDH_βE _and the critical role PDH plays in the regulation of myocardial glucose oxidation, we endeavored to better characterize the two molecular forms of PDH_βE1_. The major difference between the two forms of PDH_βE1 _is a novel O-palmitoylation PTM to the more acidic form of PDH_βE1_. In addition, I/R triggered phosphorylation of both forms of PDH_βE1 _with *in silico *methods identifying insulin receptor kinase and PKC_ζ _or PKA as the putative kinases the more basic form and acid form respectively.

The mitochondrial PDH complex catalyzes the decarboxylation of pyruvate to acetyl-CoA, and consists of multiple copies of three enzymes (E1 (pyruvate dehydrogenase), E2 (dihydrolipoyl transacetylase) and E3 (dihydrolipoyl dehydrogenase), with the E1 being a tetramer consisting of 2α and 2β subunits. This complex is regulated at multiple levels including allosterically by the substrates and products of the reaction [31] and due to phosphorylation/dephosphorylation of theα chains by the PDK and PDP [32]. Although extensive work has been performed correlating phosphorylation of PDH by the PDKs with activity and pyruvate flux [32], to our knowledge no one has previously reported phosphorylation of PDH_βE1_. The present study identified novel phosphorylation events on PDH_βE1 _that may account for the 35% decrease in glucose oxidation during reperfusion, and sustain inactivation of PDH [33,34]. However it is not known what direct effect these phosphorylation events have on the PDH complex and its activity. *In silico *methods identified PKC_ζ _or PKA as putative kinases responsible for phosphorylating the more acidic form and insulin receptor kinase was identified for the more basic form. Although little evidence connects PKC_ζ _with the regulation of cardiac energy metabolism, several reports have established a role for PKC_δ _[34-36], with the loss of PKC_δ _expression resulting in an increase in the ratio of lipid to glucose metabolites and impaired cardioprotection due to ischemic preconditioning [35,37]. Translocation of PKC_δ _to the mitochondria results in differential expression of the PDH complex and the direct binding of PKC_δ _to PDK2 can promotes phosphorylation-dependent inhibition of PDH [34,36]. The present study would suggest that PKC might also directly phosphorylate PDH_E1β _to exert its effects on PDH activity. An alternative hypothesis would be that phosphorylation at this site by PKA stimulates PDH activity, as previous reports suggest that treatment of isolated hearts with cAMP analogues or epinephrine (which signals via cAMP) preferentially increase glucose oxidation via an activation of PDH [38,39]. The phosphorylation-induced activation of PDH by insulin receptor kinase would also be consistent with the activation of glucose oxidation by insulin [40]. Athough there is little evidence implicating insulin in directly regulating PDH activity, there is some suggestion that insulin may activate PDH phosphatase 2, leading to a dephosphoryation and activation of PDH [41]. Five hours of *in vivo *insulin infusion is sufficient to decrease mRNA of PDK4, however it is unknown if this occurs with acute insulin administration [42]. The identification of these novel phosphorylation sites on PDH_βE1 _may have very important implications on the regulation of PDH activity; therefore further studies are required to determine the ability of the putative kinases to phosphorylate these sites *in vitro *and *in vivo *and their subsequent effect on PDH activity.

The *in silico *analysis also identified a novel O-palmitoylation PTM of the more acidic form of PDH_βE1_, which was confirmed biochemically. To our knowledge this is the first report of this modification in a mitochondrial protein. This finding may be of functional significance, as only protein levels of the O-palmitoylated form of PDH_βE1 _are decreased during I/R in the heart. Very few studies have reported the existence of O-acylated peptides, with one reporting that the removal of O-palmitoylation from a threonine residue in the insect toxin PLTX-II results in significant reduction in biological activity and more recently an O-octanylation of a serine residue in ghrelin was identified to be essential for its biological activity [43,44]. Due to the scarcity of O-acylation of peptides, no work has been performed on its biological role, however much is known about the S- and N-palmitoylation of proteins [45]. These PTMs tend to promote both membrane association of soluble proteins and targeting of proteins to lipid rafts. As PDH is reported to be localized to the inner mitochondrial membrane in cardiac tissue [46], we hypothesize that palmitoylation of PDH results in its recruitment to the inner mitochondrial membrane, whereby it could interact with the monocarboxylate transport (the transporter required for pyruvate entry into the mitochondria). In this case a reduction in the palmitoylated form of PDH during I/R would result in reduced inner mitochondrial membrane localization of PDH, potentially reducing pyruvate flux and glucose oxidation in the reperfused heart. Further studies are required to assess the effects of palmitoylation on PDH activity, in order to determine its effects on glucose oxidation in the ischemic heart.

A potential limitation of the present study is that our global I/R protocol is mainly a model of myocardial stunning without significant necrosis and apoptosis, which has limited clinical applicability. In addition, only subsarcolemmal mitochondria were isolated using our protocol, as nagarse was not included in our isolation buffer. This protease was omitted because it has been demonstrated that it affects the activity of NAD^+ ^glycohydrolase and the rate of NADH hydrolysis, which could potentially modify other metabolic function [47,48]. To our knowledge no one has examined the difference in the proteome of subsarcolemmal and interfibrillar mitochondria, therefore it is unknown how this would affect the interpretation of the current data. Although we identified a number of changes in mitochondrial protein abundance, the mechanism by which I/R induces these changes is unknown, but may include the release of mitochondrial proteins [8,49], 2) turnover of these proteins (balance between gene expression/protein synthesis and protein degradation) [50-52] and 3) post-translational modifications that significantly change the pI or MW of the protein [6].

## Conclusions

Using a functional proteomics approach we have identified novel changes in mitochondrial protein abundance and PTMs of metabolic proteins in response to I/R. We identified and partially characterized two molecular forms of PDH_βE1_, which differ due to a novel O-palmitoylation PTM. Also, for the first time we show that I/R induces phosphorylation of PDH_βE1_, which is associated with a decrease in glucose oxidation during reperfusion. As PDH is the rate-limiting enzyme for glucose oxidation, the regulation of these beta chain modifications may have important implications on the regulation of glucose oxidation in the heart.

## Abbreviations

ATP: adenosine triphosphate; I/R: Ischemia/reperfusion; NADH: Nicotinamide adenine dinucleotide; PDH: pyruvate dehydrogenase; PKA: Protein Kinase A; PKC: Protein kinase C; PTM: post-translational modification.

## Competing interests

The authors declare that they have no competing interests.

## Authors' contributions

CDLF participated in the study design, performed the isolated rat heart perfusions, performed data and statistical analysis and drafted the manuscript. GS participated in study design, performed the 2-D gel electrophoresis and MS identification of proteins and drafted the manuscript. GM collected the rates of metabolic fluxes in isolated hearts. AJB performed the mitochondrial isolation and revised the manuscript. VJJC performed mitochondria preparation and immunoblot analysis for purity of mitochondria preparation. GDL participated in the study design and drafted the manuscript. All authors have read and approved the manuscript.
